# Correction to “GB_SynP: A Modular dCas9-Regulated Synthetic Promoter Collection for Fine-Tuned Recombinant Gene Expression in Plants”

**DOI:** 10.1021/acssynbio.2c00542

**Published:** 2022-11-23

**Authors:** Elena Moreno-Giménez, Sara Selma, Camilo Calvache, Diego Orzáez

There are some errors in Table S3 regarding the descriptions of DNA parts GB2034-GB2039, and the missing entries for GB1724, GB1838, GB1839 and GB2033. The correct entries for these DNA parts are shown in Table S3 below. These mistakes do not affect the main text of the article.

**Table S3:** 

GB Code	Part Name	Description
GB1838	Alpha1 1gRNA-DFR 2.1 (gRNA1)	GB-cassette for the expression of guide RNA1 targeting the DFR promoter in −150 position.
GB1839	Alpha1 5gRNA-DFR 2.1 (gRNA2)	GB-cassette for the expression of guide RNA2 targeting the DFR promoter in −376 position.
GB1724	Alpha1 gRNA-Pnos:2.1 (gRNA3)	GB-cassette for the expression of guide RNA3 targeting the NOS promoter.
GB2033	Alpha1:1gRNA SlDFR	Multiplex construct to target SlDFR in −100 position.
GB2034	Alpha1:2gRNA SlDFR	Multiplex construct to target SlDFR in +31 position.
GB2035	Alpha1:3gRNA SlDFR	Multiplex construct to target SlDFR in −1320 position.
GB2036	Alpha1:4gRNA SlDFR	Multiplex construct to target SlDFR in −1052 position.
GB2037	Alpha1:5gRNA SlDFR	Multiplex construct to target SlDFR in −979 position.
GB2038	Alpha1:6gRNA SlDFR	Multiplex construct to target SlDFR in −683 position.
GB2039	Alpha1:7gRNA SlDFR	Multiplex construct to target SlDFR in −1348 position.

